# Applicability of duplex real time and lateral flow strip reverse-transcription recombinase aided amplification assays for the detection of Enterovirus 71 and Coxsackievirus A16

**DOI:** 10.1186/s12985-019-1264-z

**Published:** 2019-12-30

**Authors:** Xin-na Li, Xin-xin Shen, Ming-hui Li, Ju-ju Qi, Rui-huan Wang, Qing-xia Duan, Rui-qing Zhang, Tao Fan, Xue-ding Bai, Guo-hao Fan, Yao Xie, Xue-jun Ma

**Affiliations:** 1 NHC Key Laboratory of Medical Virology and Viral Diseases, Chinese Center for Disease Control and Prevention, National Institute for Viral Disease Control and Prevention, No.155 Changbai Road, Changping district, Beijing, 102206 China; 2 Department of hepatology Division 2, Beijing Ditan Hospital, Capital Medical University, Jing Shun Dong Jie 8#, Beijing, 100015 China

**Keywords:** Enterovirus 71, Coxsackievirus A16, Hand foot and mouth disease, Duplex, Reverse-transcription recombinase aided amplification assays, Internal amplification controls (IAC), Lateral flow strip

## Abstract

**Background:**

Enterovirus 71 (EV71) and coxsackievirus A16 (CA16) are the two main etiological agents of Hand, Foot and Mouth Disease (HFMD). Simple and rapid detection of EV71 and CA16 is critical in resource-limited settings.

**Methods:**

Duplex real time reverse-transcription recombinase aided amplification (RT-RAA) assays incorporating competitive internal amplification controls (IAC) and visible RT-RAA assays combined with lateral flow strip (LFS) for detection of EV71 and CA16 were developed respectively. Duplex real time RT-RAA assays were performed at 42 °C within 30 min using a portable real-time fluorescence detector, while LFS RT-RAA assays were performed at 42 °C within 30 min in an incubator. Recombinant plasmids containing conserved VP1 genes were used to analyze the sensitivities of these two methods. A total of 445 clinical specimens from patients who were suspected of being infected with HFMD were used to evaluate the performance of the assays.

**Results:**

The limit of detection (LoD) of the duplex real time RT-RAA for EV71 and CA16 was 47 copies and 38 copies per reaction, respectively. The LoD of the LFS RT-RAA for EV71 and CA16 were both 91 copies per reaction. There was no cross reactivity with other enteroviruses. Compared to reverse transcription-quantitative PCR (RT-qPCR), the clinical diagnostic sensitivities of the duplex real time RT-RAA assay were 92.3% for EV71 and 99.0% for CA16, and the clinical diagnostic specificities were 99.7 and 100%, respectively. The clinical diagnostic sensitivities of the LFS RT-RAA assay were 90.1% for EV71 and 94.9% for CA16, and the clinical diagnostic specificities were 99.7 and 100%, respectively.

**Conclusions:**

The developed duplex real time RT-RAA and LFS RT-RAA assays for detection of EV71 and CA16 are potentially suitable in primary clinical settings.

## Background

Hand, foot and mouth disease (HFMD) is a common acute infectious disease with typical rash distribution characteristics in the mouth, hands and feet, and occurs mainly in the children under 5 years of age. Enteroviruses such as enterovirus 71 (EV71), coxsackievirus A16 (CA16), coxsackievirus A6 (CA6) and coxsackievirus A10 (CA10) are the main pathogens causing the disease. According to previous monitoring reports, EV71 and CA16 have co-circulated as two most frequent EV types in causing repeated HFMD outbreak in different areas [1–6]. EV71-related HFMD can be accompanied by serious complications, such as myocarditis, pulmonary edema, aseptic meningitis, a proportion of which are fatal [7, 8], while CA16-related HFMD is usually mild and self-limiting.

Virus isolation, neutralization tests and nucleic acid amplification are commonly used for the detection and diagnosis of EV71 and CA16 [9, 10]. Virus isolation and neutralization are not rapid and accurate enough because of complex procedures and low specificities and sensitivities. Quantitative PCR (qPCR), reverse transcription-quantitative PCR (RT-qPCR) as a gold standard method is widely used to detect pathogens [11–17], since it is highly sensitive and specific analysis. Nevertheless, RT-qPCR protocols require specialized PCR machines and take more than 2 h. In recent years, many isothermal methods have emerged, such as nucleic acid sequence based amplification [18, 19], loop mediated isothermal amplification [20, 21], and recombinase polymerase amplification [22]. These assays are performed at a constant temperature for less than 1 h with high sensitivities, specificities and do not require use of thermal cycler, which represents valuable alternatives to carry out simple and rapid pathogen detection.

**Fig. 1:**
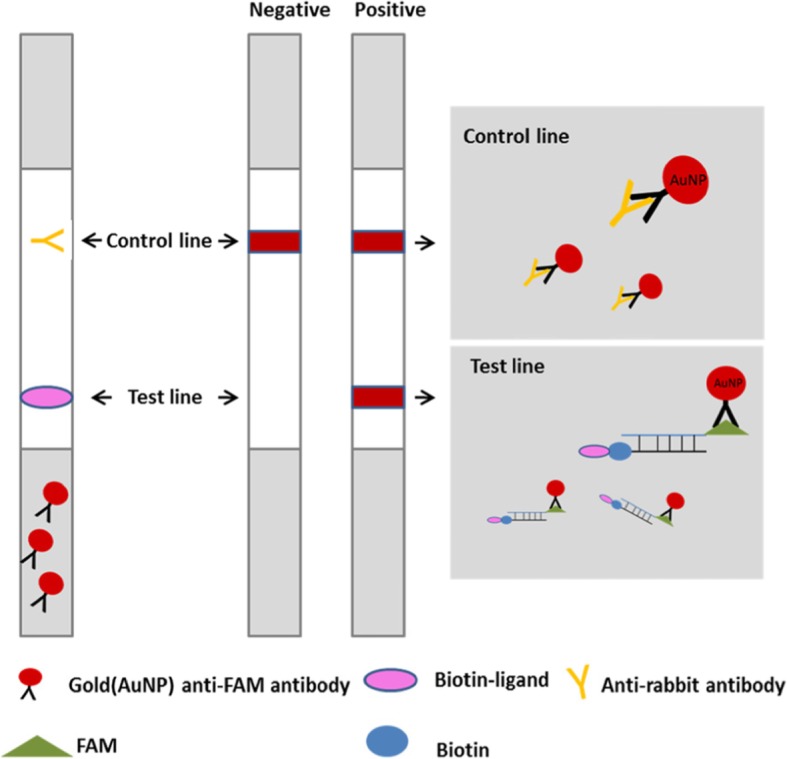
Detection of the RAA amplicons by lateral flow strip. The sample pad contains gold-labeled anti-FAM antibodies, the test line was coated with biotin-ligands, and control line was coated with anti-rabbit antibodies. The double-labeled amplicons (FAM and biotin) were diffused through the chromatographic membrane, and when they diffused to the test line, the products were captured by the biotin -ligands, resulting in an appearance of red-pink color. Non-captured particles will be fixed at the control line by anti-rabbit antibodies. In the absence of target amplicons, color will appear at control line only [26]

Recombinase aided amplification (RAA) is an isothermal amplification technique and is performed at 37–42 °C for 30 min. There are three main proteins in the RAA system: recombinase, single-stranded DNA binding protein (SSB) and DNA polymerase Klenow fragment. The amplification is initiated by a primer recombinase complex. The complex invades the DNA double strand at the homologues sequences of the primer, where SSB stabilizes the reaction. The polymerase is responsible for extension. The RAA assay can also use reverse transcriptase for the detection of RNA template. Real time detection of the RAA products can be achieved by adding exonuclease III (exo) and exo-probe. While visual detection can be realized by combining lateral flow strip (LFS) with RAA assay, making it an ideal technique for point-of-care testing [23–25]. The expected result of the positive reaction is clear colored test line and control line on the strip. The negative reaction does not generate a signal at the position of the test line (Fig. 1) [24].

Previous studies reported on the applications of RAA in the detection of salmonella [27] and RSV, CA6 and CA10 and HBV [28–30], but these methods did not use internal amplification control (IAC) or LFS. The objective of the research is to establish sensitive and rapid RAA assays for the detection of EV71 and CA16, including duplex real time RT-RAA assay containing IAC to reduce the false negative rates and LFS RT-RAA assay suitable for field detection in resource limited areas.

## Methods

### Clinical samples

HFMD clinical diagnostic criteria was referred to Hand-foot-mouth disease diagnosis and treatment guidelines (2010 version) [31]. A total of 445 clinical samples from patients (7 months to 11 years of age) who were suspected of being infected with HFMD in Shandong province, Hebei province, and Hunan province in China were collected during the period from January 2016 to December 2016 for this study. Sample types included throat swabs (*n* = 76), anal swabs (*n* = 25) and stools (*n* = 344). Ethics approval was granted by the local ethics committee.

### Nucleic acid extraction

Pre-treatment of clinical specimens was described previously [30]. According to the instructions recommended by the manufacturer, the total RNA was extracted from 200 μL of sample preservation solutions or supernatants (fecal treatment fluid) using the Tian Long RNA extraction kit (Tian Long, China). The nucleic acid was eluted in 100μLof nuclease free water.

### Preparation of plasmid standards and IAC plasmids

**Fig. 2:**
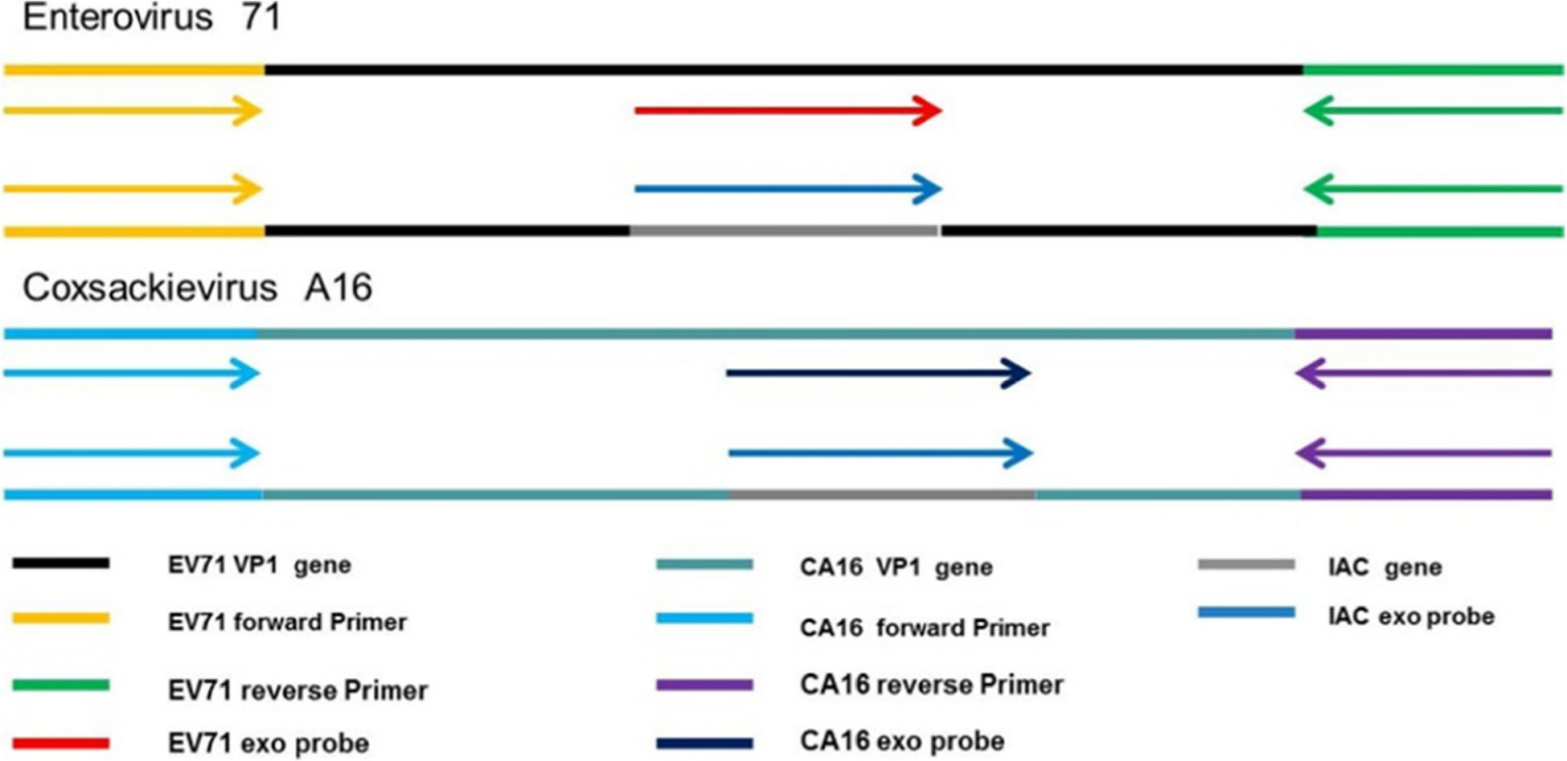
Schematic diagram of duplex real time RAA assays for detection of EV71 and CA16

The cDNA of the viral protein 1 gene of EV71 or CA16 was cloned into the pClone007 vector, the standard recombinant plasmids with 10-fold concentrations ranging from 10^6^ copies /μL to10^0^ copies /μL were made and stored at − 80 °C until used. The IAC templates were recombinant plasmids consisting of the IAC exo probe sequence, a short gene sequence of rose rosette virus [32], which replaced the corresponding probe sequences of EV71 and CA16, respectively (Fig. 2).

### Primers and probes design of RT-RAA assays for the detection of EV71 andCA16

The VP1 genes of both EV71 and CA16 were chosen as the targets because VP1 was a specific region for enterovirus genotyping. All the available VP1 genes of EV71 and CA16 were downloaded from GenBank database. The sequences were aligned by Unipro UGENE. The primers were manually designed following the principle of RAA primer and probe design [33]: (1) Primers usually are 30–35 nucleotides long in length. (2) Long tracks of guanines at the 5′ end are avoided. (3) Guanines and cytidines at the 3′ end are preferred. (4) Probes are about 46–52 nucleotides long in length. (5) A DNA exo probe is used for real time assay, consisting of an oligonucleotide with homology to the target amplicon and an abasic nucleotide analogue (tetrahydrofuran residue or THF) flanked by a dT-fluorophore and a corresponding dT-quencher group. In addition, probe is blocked from polymerase extension by C3-spacer. (6) A DNA probe is used for LFS assay, consisting of 5-FAM antigenic labeled upstream stretch (30 nucleotides) connected via THF spacer to an adjacent downstream oligonucleotide (15 nucleotides) carrying a C3-spacer. (7) Reverse primers used for LFS assay is labelled at its 5′ end with biotin. The target exo probes and IAC exo probe were modified with FAM and HEX fluorophores, respectively. All the primer and probe sequences are listed in Table 1. The primers and probes were synthesized by Sangon Biotech (Shanghai, China).

**Table 1:** List of primers used in the study for rapid detection of EV71 and CA16

Assay	Primer/probe	Sequence 5′- 3′	Genomic Position ^a^	Product size
Real time RAA	EV71-exo-F	CCTGCGAGTGCTTACCAATGGTTTTATGACGG	3026–3057	199 bp
	EV71-exo-R	GTATCCACGCCCTGACGTGCTTCATTCTCAT	3194–3224	
	EV71-exo-P	AACATGATGGGCACGTTCTCAGTGCGGAC-[FAM-dT]-[THF] -[BHQ-dT]-GGGGACCTCCAAGTC-C3-spacer	3119–3165	
	CA16-exo-F	GCAAGTAGTCACAGATTAGGCACTGGTGTTGT	2557–2588	161 bp
	CA16-exo-R	GCACGGCTAAAGAAATTCCCAATGGCTGTC	2688–2717	
	CA16-exo-P	GTGACAAGAATCTCATTGAGACKAGATG-[FAM-dT]-[THF] [BHQ-dT]-GTTGAACCATCACTCCA-C3-spacer	2633–2680	
	IAC-P	GTAAGGTGCTAGACTAAAATTGTTGGGACTT- [HEXdT]-G [THF]-A-[BHQ-dT]-CTCTGAAGTAAAAGG-C3-spacer		
LFS RAA	EV71-LF-F	CCTGCGAGTGCTTACCAATGGTTTTATGACGG	3026–3057	199 bp
	EV 71-LF-R	Biotin-GTATCCACGCCCTGACGTGCTTCATTCTCAT	3194–3224	
	EV71-LF-P	FAM-AACATGATGGGCACGTTCTCAGTGCGGAC -[THF]-TGGGGACCTCCAAGTC-C3-spacer	3119–3165	
	CA16-LF-F	GCAAGTAGTCACAGATTAGGCACTGGTGTTGT	2557–2588	161 bp
	CA16-LF-R	Biotin-GCACGGCTAAAGAAATTCCCAATGGCTGTC	2688–2717	
	CA16-LF-P	FAM -GTGACAAGAATCTCATTGAGACKAGATG-[THF] -TGTTGAACCATCACTCCA-C3-spacer	2633–2680	

^a^Genome position depending on Enterovirus A71 strain HP (GenBank accession no.KY074643.1) and Coxsackievirus A16 isolate ZJ10–48 (GenBank accession no. KC755235.1)

### Duplex real time RT-RAA assay

The duplex real time RT-RAA assays were carried out using the exo-RT-RAA lyophilized kit (Qitian Bio-Tech, China). The total reaction volume was 50 μL containing 420 nM of RAA primers, 120 nM of target exo probe for EV71, or 150 nM of target exo probe for CA16, and 60 nM of IAC exo probe for IAC, and an IAC recombinant plasmid (50 copies per reaction for EV71 or100 copies per reaction for CA16, respectively),14 mM magnesium acetate and 2 × buffer. The RT-RAA reagents were made in a master mix and were rehydrated pellets. Magnesium acetate and 5 μL of template were added into the reaction tubes, subsequently, tubes were placed into B6100 Oscillation mixer (QT-RAA-B6100, Jiangsu Qitian Bio-Tech Co. Ltd., China) and incubated for 4 min, then mixed and centrifuged briefly, transferred to fluorescence detector (QT-RAA-1620, Jiangsu Qitian Bio-Tech Co. Ltd., China) at 42 °C for 30 min. The FAM channel was used to detect the amplification of the target gene (EV71 or CA16), and the HEX channel was used to detect the amplification of the IAC. Fluorescence data were normalized and baseline was adjusted using RAA 1620 software. External positive and negative controls were included to avoid false negative and positive results in each assay.

### LFS RT-RAA assay and optimization of the reaction time

LFS RT-RAA assays were performed using the nfo-RT-RAA lyophilized kit (Qitian Bio-Tech, China). The total 50μLvolume containing 420 nM of RAA primers, 120 nM of LF probe for EV71, 150 nM of LF probe for CA16. To determine the optimal time of the LFS RT-RAA assays, the tubes were incubated in the pre-equilibrated device at 42 °C for 10, 20, 30 and 40 min, respectively, using 1.0 × 10^2^ copies of the recombinant plasmids as templates. After amplification, the RAA products were detected by lateral flow strip (Ustar Biotechnologies, Hangzhou, China) according to the instruction of manufacturer. The result was considered negative if only the control line was visible. The result was considered to be positive when both the control line and test line visible (Fig. 1).

### Analytical sensitivity and specificity of duplex real time RT-RAA and LFS RT-RAA assays

The analytical sensitivity of real time RT-RAA and LFS RT-RAA assays for EV71 and CA16 were tested by using a serial dilution of recombinant plasmid standards ranging from 10^6^ to 10^0^copies in eight replicates. The LoD of two assays were calculated using the probit regression analysis with the SPSS version 17.0. The analytical specificity of the RT-RAA assays for EV71 and CA16 was respectively tested by using 170 Non-EV71, non-CA16 enterovirus positive specimens (out of 445) in the study.

### Evaluation of duplex real time RT-RAA and LFS RT-RAA with clinical specimens

The duplex real-time and LFS RT-RAA assays were assessed by using 445 clinical specimens. For the duplex real time RT-RAA assays, detection results were considered positive by simultaneously generating amplification curves from both clinical specimens and IAC. Detection was identified to be negative when there was only amplification of IAC. Neither IAC nor clinical specimens was amplified indicated invalid assay. For the LFS RT-RAA assays, if the control line and test line appeared simultaneously, the result was judged to be positive; if there was only control line, it was judged to be negative, if there was no control line and test line, it was judged to be invalid. The real time RT-qPCR assays for EV71, CA16 and other enteroviruses were carried out simultaneously as parallel tests [11]. The result was judged as positive when threshold cycle (Ct) value was less than 35. Inconsistent detection results were further resolved using nested RT-PCR assays and sequencing [34]. The overall clinical performance was evaluated by calculating diagnostic sensitivity, diagnostic specificity, positive predictive value (PPV), negative predictive value (NPV), and Kappa value [35, 36].

## Results

### Analytical sensitivity and specificity of duplex real time RT-RAA assay

**Fig. 3:**
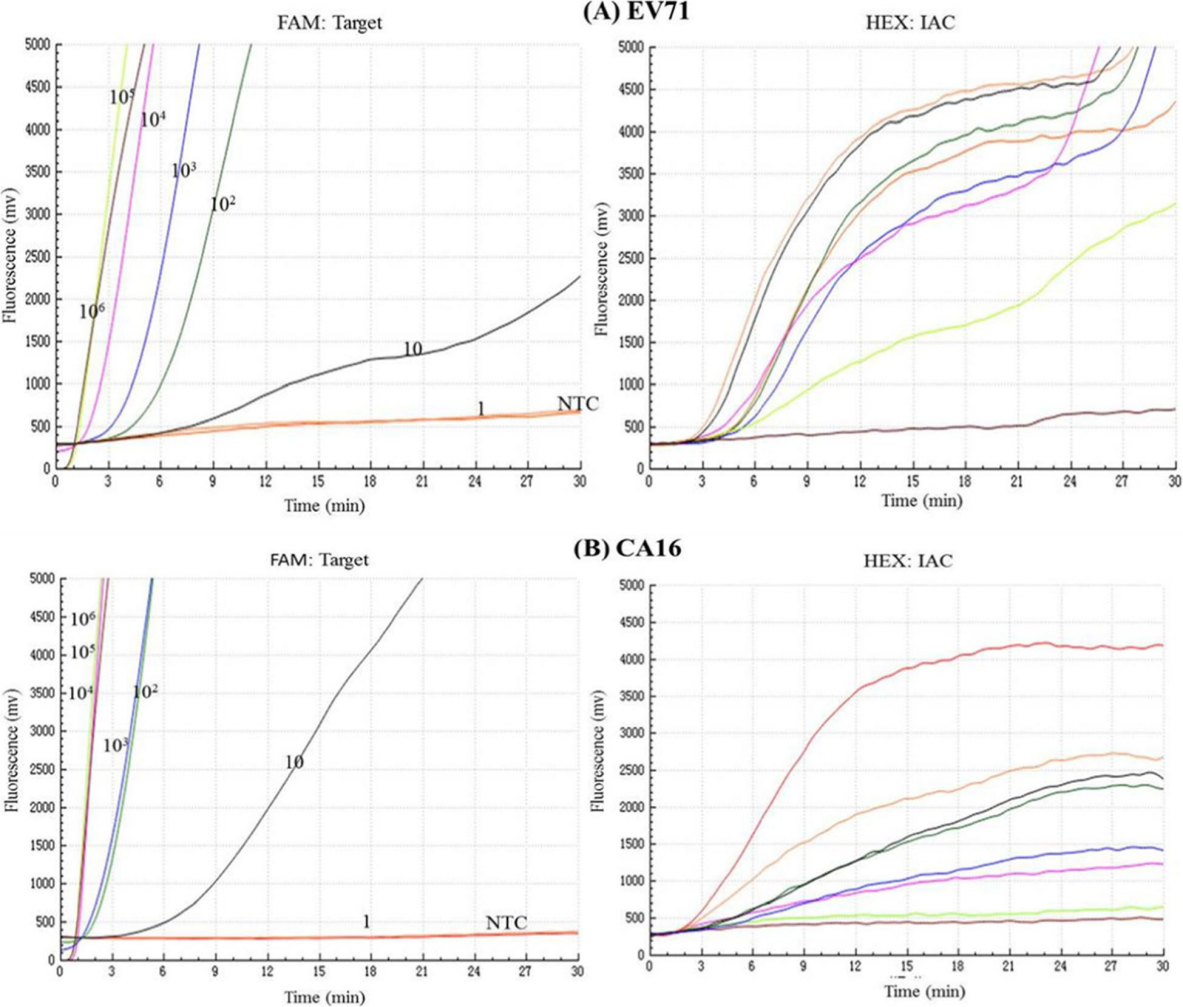
The amplification curves of the duplex real time RT-RAA assays using 10-fold dilution series of plasmid DNA containing the VP1gene of EV 71 (**a**) and CA16 (**b**). Fluorescence signals from target amplification were recorded in the FAM detection channels of the QT-RAA1620 device, while IAC amplification was recorded in the HEX detection channel. The development of fluorescence using a dilution range of 10^6^ copies/μL − 1 copy/μL of the target recombinant plasmid standards. **a** represents the duplex RT- RAA of EV71 with 50copies IAC plasmid per reaction. The sensitivity of the assay is 10 copies. High concentrations of target plasmids (10^6^copies/μL) completely inhibit the amplification of IAC plasmid. EV71 amplification curves of high concentration plasmid (10^6^ copies per reaction) occurred early and even overlapped, while for the IAC, no amplification curves were observed in the HEX channel. EV71 amplification curves of medium and low concentration plasmids appeared slightly later than the high concentration, while for the IAC, amplification curves were observed in the HEX channel. **b** represents the duplex RT-RAA of CA16 with 100 copies IAC plasmid per reaction. The sensitivity of the assay is 10 copies. High concentrations of target plasmids (10^6^-10^5^copies/μL) completely inhibit the amplification of IAC plasmid. The amplification curves of CA16 plasmids with high concentration (10^6^ -10^5^copies per reaction) appeared early and even overlapped, while for the IAC, no amplification curves were observed in the HEX channel. CA16 plasmids with medium and low concentrations revealed no significant interference with IAC amplification curves in the HEX channel

As shown in the Fig. 3, in the FAM channel, increase of fluorescence signal was observed from 1× 10^6^ to 1 × 10^0^ copies/reaction at 42 °C within 30 min. In the HEX channel, IAC was well amplified in the presence of low concentration of target (<10^3^copies), while inhibited in the presence of high concentration of target (>10^5^copies). Both EV71 and CA16 duplex real time RT-RAA assays were able to detect 10 copies per reaction in the presence of 50 and 100 copies IAC plasmids, respectively. The LoD of EV71and CA16 duplex real time RT-RAA at 95% probability was 47 copies/reaction and 38 copies/reaction, respectively (Table 2). No cross-reactivity was observed with other 170 non-EV71, non-CA16 clinical specimens.

**Table 2:** Assay data used for calculating the detection limit of EV71 and CA16

Standard DNA^a^ (copies/reaction)	EV71pos^b^		CA16pos^b^	
	Real time RAA	LFS RAA	Real time RAA	LFS RAA
10^4^	8/8	8/8	8/8	8/8
10^3^	8/8	8/8	8/8	8/8
100	8/8	8/8	8/8	8/8
20	5/8	0/8	6/8	0/8
10	3/8	0/8	4/8	0/8
1	0/8	0/8	0/8	0/8

^a^Tenfold serially diluted standard DNA

^b^The number of positive results per 8 reactions with diluted standard DNA

### Optimization of the reaction time, analytical sensitivity and specificity of LFS RT-RAA assay

**Fig. 4:**
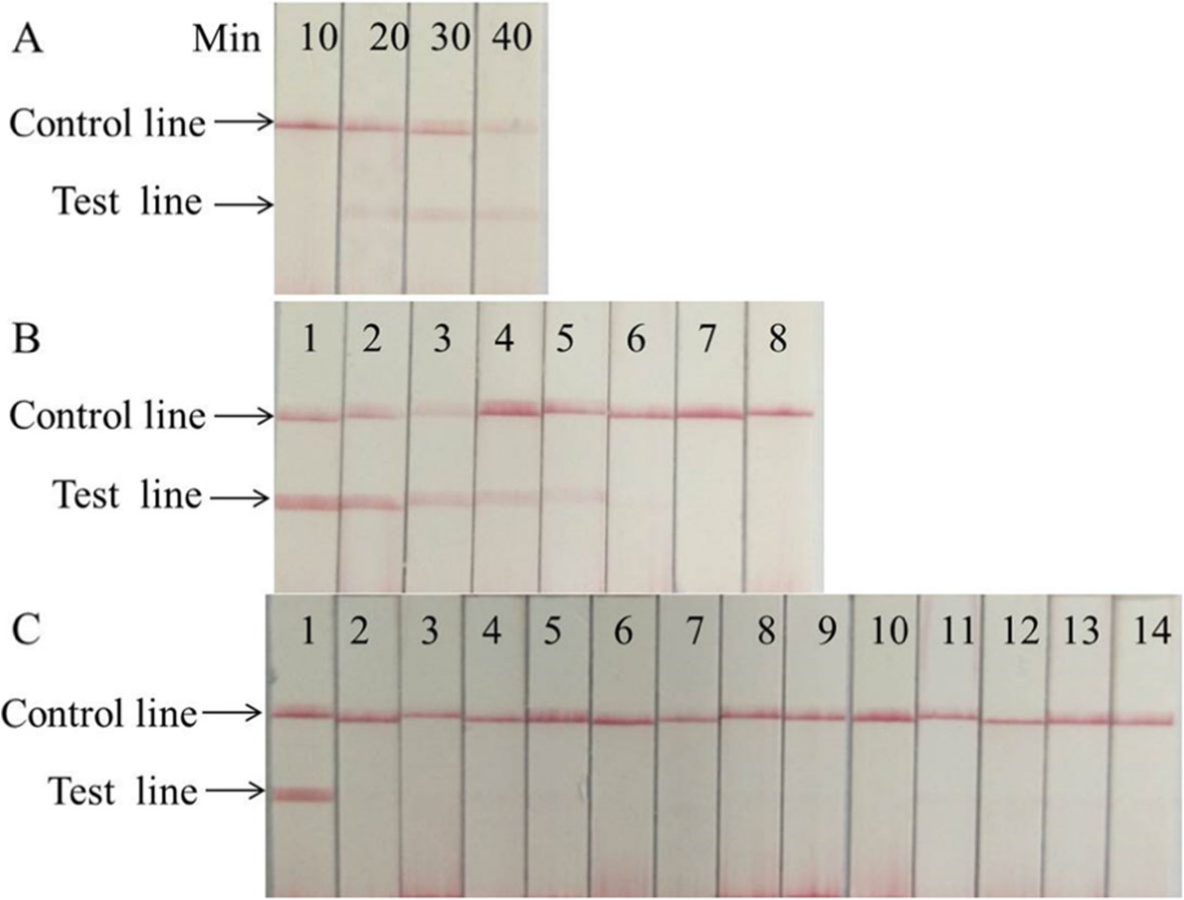
Performance of EV71 LFS RT-RAA assay. **a** Optimization experiment of LFS RT-RAA reaction time. When the reaction time was longer than 20 min, the test line was visible. **b** Analytical sensitivity of the LFS RT- RAA assay. Lane1, 1 × 10^6^ copies, Lane2, 1 × 10^5^copies, Lane3, 1 × 10^4^copies, Lane4, 1 × 10^3^copies, Lane5, 1 × 10^2^ copies, Lane6, 1 × 10^1^ copies, Lane7, 1 copy, Lane8, no template control. **c** Analytical specificity of the LFS RT-RAA assay. Only EV71 specimen was amplified. The other samples were not amplified. Lane1, EV71, Lane2, CA 16, Lane3, CA6, Lane4, CA10, Lane5, CA5, Lane6, CA9, Lane7, CA24, Lane8, CB2, Lane9, CB4, Lane10, PV2, Lane11, PV3, Lane12, Eco30, Lane13, HEV14, Lane14, no template control

**Fig. 5:**
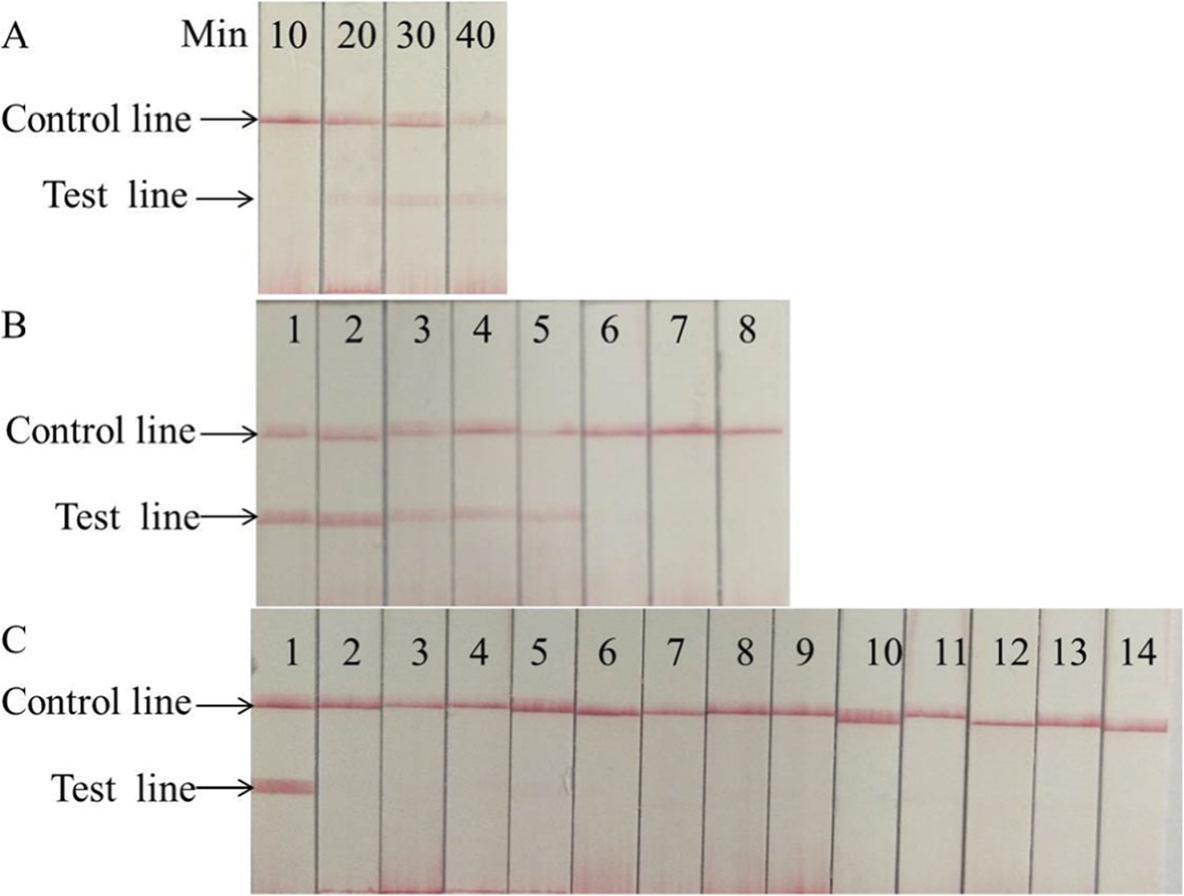
Performance of CA16 LFS RT-RAA assay. **a** Optimization experiment of LFS RT-RAA reaction time. When the reaction time was longer than 20 min, the test line was visible. **b** Analytical sensitivity of the LFS RT-RAA assay. Lane1, 1 × 10^6^ copies, Lane2,1 × 10^5^copies, Lane3, 1 × 10^4^copies, Lane4, 1 × 10^3^copies, Lane5, 1 × 10^2^ copies, Lane6, 1 × 10^1^ copies, Lane7, 1copy, Lane8, no template control. **c** Analytical specificity of the LFS RT-RAA assay. Only CA16 specimen was amplified. The other samples were not amplified. Lane1, CA16, Lane2, EV71, Lane3, CA6, Lane4, CA10, Lane5, CA5, Lane6, CA9, Lane7, CA24, Lane8, CB2, Lane9, CB4, Lane10, PV2, Lane11,PV3, Lane12, Eco30, Lane13, HEV14, Lane14, no template control

As shown in Fig. 4a (EV71) and Fig. 5a (CA16), no test line was observed for the 10 min incubation, and the test line was weakly visible for 20 min incubation using 100copies plasmid as a template, and no clear difference was shown between 30 min and 40 min incubation. Therefore, the reaction time was chosen to be 30 min.

As shown in Fig. 4b and Fig. 5b, the results indicated that the sensitivities of the LFS RAA assays for EV71 and CA16 were100 copies per reaction. The detection limits of LFS RT-RAA for EV71 and CA16 were both 90 copies. For the specificity analysis, only EV71 or CA16 was detected by the LFS RT-RAA assays, not other control enteroviruses. These control viruses included coxsackievirus A6 (CA6), coxsackievirus A10 (CA10), coxsackievirusA5 (CA5), coxsackievirus A9 (CA9), coxsackievirus A24 (CA24), coxsackievirus B2 (CB2), coxsackievirus B4 (CB4), poliovirus 2 (PV2), poliovirus 3 (PV3), echovirus 30 (Eco30), and human enterovirus 14 (HEV14) (Fig. 4c, Fig. 5c)

### Evaluation of duplex real time RT-RAA and LFS RT-RAA assays with clinical specimens

A total of 445 suspected HFMD specimens were used for the clinical evaluation of duplex real time RT-RAA and LFS RT-RAA assays for EV71 and CA16.The RT-qPCR assays were carried out simultaneously as parallel tests. RT-qPCR results indicated that 20.4% (91/445), 22.0% (98/445), 38.2% (170/445) of the specimens were EV71, CA16 and other enterovirus positive. The remaining 86 samples were RT-qPCR negative, as no enterovirus was identified in these samples.

As shown in the Table 3, with RT-qPCR as the reference assay, the diagnostic sensitivities of duplex real time and LFS RT-RAA assays for EV71 were 92.3, 90.1%, respectively, the diagnostic specificities were both 99.7%, the positive predicative values were both 98.8%, the negative predictive values were 98.1, 97.5%, respectively. Both assays had high consistency (Kappa values: 0.943, 0.929). The diagnostic sensitivities of duplex real time and LFS RT-RAA assay for CA16 were 99.0, 94.9%, respectively, the diagnostic specificities and the positive predicative values were both 100%, the negative predictive values were 99.7, 98.6%, respectively. Both assays also had high consistency (Kappa values: 0.993, 0.967). As to the samples with discrepant detection results, 7 EV71 specimens and 1 CA16 sample missed by duplex real time RT-RAA were found to be positive by RT-qPCR, 9 EV71 specimens and 5 CA16 samples missed by LFS RT-RAA were found to be positive by RT-qPCR. These samples were later confirmed by Sanger sequencing to be true positives. Besides, 1 EV71 positive specimen by duplex real time RT-RAA was tested negative by RT-qPCR, which was later confirmed to be true positive by sequencing.

**Table 3:** Comparison of clinical evaluation of two RT-RAA assays and RT-qPCR assay to detect EV71 and CA16

Virus	RT- qPCR	Duplex real time RT-RAA		LFS RT-RAA	
		Positive	Negative	Positive	Negative
EV71					
Positive		84	7	82	9
Negative		1	353	1	353
Diagnostic sensitivity (%)		92.3		90.1	
Diagnostic specificity (%)			99.7		99.7
PPV (%)		98.8		98.8	
NPV (%)			98.1		97.5
Kappa		0.943		0.929	
CA16					
Positive		97	1	93	5
Negative		0	347	0	347
Diagnostic sensitivity (%)		99.0		94.9	
Diagnostic specificity (%)			100		100
PPV (%)		100		100	
NPV (%)			99.7		98.6
Kappa		0.993		0.967	

*PPV* Positive predictive value, *NPV* Negative predictive value

## Discussion

Currently, HFMD is a serious threat to the health of children in China. In the study, we established and evaluated duplex real time RT-RAA and LFS RT-RAA assays for the detection of EV71 and CA16, respectively. In comparison with RT-qPCR, duplex real time RT-RAA assays showed higher diagnostic sensitivities (92.3, 98.9%) than corresponding LFS RT-RAA assays (90.1, 94.9%) in detecting EV71 and CA16 respectively. The specificities of two RT-RAA assays were further confirmed by testing 170 other enterovirus RT-qPCR-positive samples. These samples included 57 of CA6, 40 of CA10, which are increasingly prevalent in causing HFMD in China. These results together demonstrated that the proposed methods reveal a high consistency with RT-qPCR method.

Seven EV71 specimens and one CA16 sample were missed by duplex real time RT-RAA, as these sample were with high CT values (> 32) by RT-qPCR. Other than the above seven EV71 specimens, two additional EV71 specimens were not detected by LFS RT-RAA. In the case of CA16 specimens, five additional specimens were not detected by LFS RT-RAA. The sequences of RT-RAA primers and probes were subsequently compared with the templates of these samples. Sequence alignment showed that there were 2–3 mismatches occurred in the middle of the forward primer, 1–3 mismatches occurred in the middle of the exo probe. We speculate that sequence variation leads to amplification failure of RT-RAA assay [37]. Previous researches have indicated that the sequences of strains prevalent in different regions were slightly different [38–40], the genotype C, subtype 4a (C4a) of EV71 and genotype B, subtypes 1a (B1a) and1b (B1b) of CA16 are the major subgenotypes in the mainland of China. Despite this, as the clinical samples in the study were collected from three different cities, one is located in Southern part, one is situated in central part and another in Northern part of China, our results indicated the adaptability of duplex RT-RAA assay for the EV71 and CA16.

We collected different types of specimens in this study, including throat swab specimens(*n* = 76), anal swab specimens (*n* = 25) and stool specimens (*n* = 344), the results showed that there was no significant difference in the detection rate of different specimen types, suggesting the method has very good practicability in testing different types of clinical specimens.

LAMP was previously reported in the detection of EV71 and CA16 [20], however, LAMP assay did not contain an IAC. RPA is increasingly used in agriculture, food safety and pathogen detection [41–45], but few publications reported duplex real time RPA assay containing IAC. Dan Yin [22] reported a rapid RT-RPA assay to detect EV71. The 95% detection limit was 3.767 log10 genomic copies (LGC)/reaction, with100% specificity, but no IAC was included in the assay. In our study, the introduction of IAC effectively eliminated false negative results or invalid results. Two strategies are used to design IAC: one is a noncompetitive system, the other is competitive system [46–49]. A noncompetitive IAC system contains 2 pairs of primers to amplify the target DNA and control DNA, respectively. The shortcoming of a noncompetitive IAC is that it might reduce amplification efficiency for target because of the introduction and interference of control primers with target primers. In the case of competitive strategy, one set of common primers is used to amplify both the target DNA and the IAC, which eliminates the risk of interference among multiple pairs of primers. By optimizing the amount of primers, the ratio of target probes and IAC probes, and the amount of IAC plasmids, the negative impact of IAC on the detection sensitivity of target could be minimized.

Although same primers and probes were used for the amplification, the LFS RT-RAA assay was less sensitive than duplex real time RT-RAA assay. As LFS RT-RAA assay is to detect the double-labeled amplicons generated by extension of a small portion of the amplification product driven by the post-cleavage LF probe and reverse primer, this may influence the sensitivity of the LFS RT-RAA assay. Another influence factor might be due to the detection principle of lateral flow strip. The minimum number of molecules that can be detected on the test strip would also affect the final test results. While the detection of real time RT-RAA mainly depends on the fluorescence device and analyze software, results are more accurate and sensitive.

The duplex real time RT-RAA assay can be completed in a single tube in one step at 42 °C within 30 min without complicated operations or expensive equipment compared to traditional real time PCR. Additionally, introduction of the IAC effectively avoided the appearance of false negatives and invalid results. Use of the B6100 Oscillation mixer helped to further reduce manual error and improve repeatability of experimental results. Although the sensitivity of LFS RT-RAA is slightly lower than the sensitivity of duplex real-time RT-RAA, equipment-free and visual detection make the LFS RT-RAA well suitable for on-site screening in resource-limited areas. There are still some limitations in this study such as slightly lower sensitivity of duplex real time RT-RAA assay and lack of IAC in LFS RT-RAA assay. For improved practicability for point-of-care testing in resource poor clinical settings, the sensitivities of both RT-RAA assays need to be further increased to meet the requirement of initial screening while maintaining appropriate specificity. Future study will also include attempting the direct RT-RAA detection without RNA extraction and clinical evaluation using large sample size.

## Conclusion

In summary, the study demonstrated that the duplex real time RT-RAA assay is rapid and sensitive enough to detect EV71 and CA16 from clinical specimens and LFS RT-RAA assay is potentially suitable for field use in primary clinical settings.

## Data Availability

The datasets used and analyzed during the current study are included within this article and in additional file.

## Abbreviations

CA10: Coxsackievirus A10
CA16: Coxsackievirus A16
CA24: Coxsackievirus A24
CA5: Coxsackievirus A5
CA6: Coxsackievirus A6
CA9: Coxsackievirus A9
CB2: Coxsackievirus B2
CB4: Coxsackievirus B4
Eco30: Echovirus 30
EV71: Enterovirus 71
HEV14: Human enterovirus 14
HFMD: Hand, Foot and Mouth Disease
IAC: Internal amplification controls
LAMP: Loop mediated isothermal amplification
LFS: Lateral flow strip
LoD: The limit of detection
NPV: Negative predictive value
PPV: Positive predictive value
PV2: Poliovirus 2
PV3: Poliovirus 3
qPCR: Quantitative PCR
RPA: Recombinase polymerase amplification
RT-qPCR: Reverse transcription-quantitative PCR
RT-RAA: Reverse-transcription recombinase aided amplification
